# Depressive symptoms and malnutrition are associated with other geriatric syndromes and increase risk for 30-Day readmission in hospitalized older adults: a prospective cohort study

**DOI:** 10.1186/s12877-022-03343-6

**Published:** 2022-08-02

**Authors:** Tay Laura, Chua Melvin, Ding Yew Yoong

**Affiliations:** 1 Department of General Medicine, Sengkang General Hospital, 110 Sengkang East Way, 544886 Singapore, Singapore; 2 Geriatric Education and Research Institute, Singapore, Singapore; 3 Department of Geriatric Medicine, Tan Tock Seng Hospital, Singapore, Singapore

**Keywords:** Depression, Malnutrition, Readmission, Older adults

## Abstract

**Background:**

Readmission in older adults is typically complex with multiple contributing factors. We aim to examine how two prevalent and potentially modifiable geriatric conditions – depressive symptoms and malnutrition – relate to other geriatric syndromes and 30-day readmission in hospitalized older adults.

**Methods:**

Consecutive admissions of patients ≥ 65 years to a general medical department were recruited over 16 months. Patients were screened for depression, malnutrition, delirium, cognitive impairment, and frailty at admission. Medical records were reviewed for poor oral intake and functional decline during hospitalization. Unplanned readmission within 30-days of discharge was tracked through the hospital’s electronic health records and follow-up telephone interviews. We use directed acyclic graphs (DAGs) to depict the relationship of depressive symptoms and malnutrition with geriatric syndromes that constitute covariates of interest and 30-day readmission outcome. Multiple logistic regression was performed for the independent associations of depressive symptoms and malnutrition with 30-day readmission, adjusting for variables based on DAG-identified minimal adjustment set.

**Results:**

We recruited 1619 consecutive admissions, with mean age 76.4 (7.9) years and 51.3% females. 30-day readmission occurred in 331 (22.0%) of 1,507 patients with follow-up data. Depressive symptoms, malnutrition, higher comorbidity burden, hospitalization in the one-year preceding index admission, frailty, delirium, as well as functional decline and poor oral intake during the index admission, were more commonly observed among patients who were readmitted within 30 days of discharge (*P* < 0.05). Patients with active depressive symptoms were significantly more likely to be frail (OR = 1.62, 95% CI 1.22–2.16), had poor oral intake (OR = 1.35, 95% CI 1.02–1.79) and functional decline during admission (OR = 1.58, 95% CI 1.11–2.23). Malnutrition at admission was significantly associated with frailty (OR = 1.53, 95% CI 1.07–2.19), delirium (OR = 2.33, 95% CI 1.60–3.39) cognitive impairment (OR = 1.88, 95% CI 1.39–2.54) and poor oral intake during hospitalization (OR = 2.70, 95% CI 2.01–3.64). In minimal adjustment set identified by DAG, depressive symptoms (OR = 1.38, 95% CI 1.02–1.86) remained significantly associated with 30-day readmission. The association of malnutrition with 30-day readmission was no longer statistically significant after adjusting for age, ethnicity and depressive symptoms in the minimal adjustment set (OR = 1.40, 95% CI 0.99–1.98).

**Conclusion:**

The observed causal associations support screening and targeted interventions for depressive symptoms and malnutrition during admission and in the post-acute period.

**Supplementary Information:**

The online version contains supplementary material available at 10.1186/s12877-022-03343-6.

## Background

Hospitalized older adults represent a medically complex population and are susceptible to unplanned early readmission [1, 2], exposing the vulnerable older person to recurrent hazards of hospitalization that negatively impact their prognosis [3]. While readmissions have often been attributed to suboptimal transitional care, many hospital-initiated transition programs had little impact on readmission rate, and effective interventions are often resource- and personnel-intensive [4, 5]. Thus, cost efficiency mandates that such multi-modal transitional care interventions be targeted towards patients at highest risk for readmission.

Risk stratification tools designed to predict readmission in older adults have demonstrated varying discriminative ability [6–10]. The inconsistent performance of existing models reiterates the need to consider utilisation outcomes and availability of data sources when considering employment of risk prediction models [11]. Most predictive models incorporate age, comorbidity and prior healthcare utilization which may not serve to further risk-stratify an already high-risk population of frail older adults with multi-morbidity. As such, identifying clinically actionable data to triage hospitalized older adults to specific foci of interventions should be considered to address the varying levels of needs. A recent study highlighted the association between geriatric syndromes and readmission within one year [12]. The inclusion of geriatric syndromes in a predictive scoring system also yielded moderate discriminative ability for 30-day readmission in older adults [13]. Geriatric syndromes are multifactorial conditions that often do not fit into discrete disease categories, share risk factors and commonly co-exist [14]. These recent findings should support comprehensive geriatric assessment (CGA) in identifying older patients at risk for readmission. However, determining the causal pathways by which individual geriatric syndromes lead to readmission may allow for more targeted interventions to address the inciting trigger, especially in high acuity settings that may be constrained by time and manpower resources for a full CGA.

Depression and malnutrition represent modifiable factors to address readmission risk, having been associated across studies with readmission outcomes although their causal pathways were not always explicitly described [13, 15–17]. Additionally, both are common risk factors for other geriatric syndromes such as frailty and cognitive impairment [18, 19], and potentially predispose to intermediary events including functional decline and poor oral intake during the index admission, all of which may further contribute to the patient’s risk for readmission. Thus, with hospital readmissions in older adults being typically complex and multifactorial, we propose the use of graphical representations of exposure-outcome relationship to facilitate analysis for a less biased estimate of the total causal effect of depression and malnutrition. Directed acyclic graphs (DAGs) have permeated epidemiology and raised awareness of the inadequacy of conventional criteria for identifying or adjusting for confounders [20]. We construct DAGs to depict how depression and malnutrition relate to each other as well as other geriatric syndromes, in their relationship with 30-day readmission outcome.

The objectives of this study were to examine (i) association between depressive symptoms and malnutrition with other geriatric syndromes, and (ii) association between depressive symptoms and malnutrition with unplanned 30-day readmission in hospitalized older adults. DAGs were applied to identify a minimally sufficient adjustment set for quantitative analyses for the magnitude of effects. We also compared the results from DAG-driven approach with the results from traditional methods of selecting variables for adjustment in multiple regression models.

## Methods

### Study setting and participants

This prospective cohort study recruited community-dwelling older adults aged ≥ 65 years who were admitted to the Department of General Medicine, Sengkang General Hospital (SKH), Singapore, over a 16-month period. All consecutive admissions during the recruitment period were screened for study eligibility. We excluded patients who were admitted from sheltered or nursing homes, direct admissions to high dependency or intensive care units at presentation, demised during hospitalization, admissions to non-medical departments, and patients who were transferred to other acute hospitals or discharged against medical advice. The first admission of unique patients to SKH between October 2018 and January 2020 was defined as the index admission.

Ethics approval for conduct of this study was obtained from SingHealth Institutional Review Board. Informed consent was obtained from all participants, or their legally acceptable representative in cases when the participant was unable to provide informed decision.

### Defining outcome

An unplanned hospital readmission was defined as at least an overnight stay in any acute hospital up to 30 days from the index discharge, irrespective of the admitting department, and that had not been previously scheduled. Observations within the Emergency Department for which an admission was not actualized were excluded, and only the first readmission was counted. Readmission data was tracked through the hospital’s electronic health records and follow-up telephone interviews were conducted to facilitate capture of readmissions to other acute hospitals that did not share the index hospital’s electronic platform.

### Clinical assessment and data collection

Table 1 summarizes the definitions and measures used for the key variables. Screening for depression was performed within 48 h of admission using the Patient Health Questionnaire-2 (PHQ-2), asking patients “Over the past 2 weeks, (i) have you felt down, depressed, or hopeless? and (ii) have you felt little interest or pleasure in doing things?” A positive response to either question was considered as presence of depressive symptomatology [21]. Medical records as well as medication list were reviewed to clarify any diagnosis of depression that had been established prior to the index admission. Patients were categorized in one of 4 groups: (i) No depressive symptoms and no history of depression (PHQ2- / History-), (ii) No depressive symptoms but positive history of depression (PHQ2- / History+), (iii) Depressive symptoms regardless of history (PHQ2+ / History±), (iv) Inability to communicate symptoms (due to reasons such as advanced dementia or hypoactive delirium). This categorization allows the differentiation of patients with active depressive symptomatology versus those whose mood has been adequately addressed, without dismissing patients who are unable to comprehend or communicate their responses to the PHQ2 as being asymptomatic.

Malnutrition was identified using the locally validated 3-minute Nutrition Screen, which was routinely administered by a trained nurse upon admission. A score ≥ 3 (range 0–9) is indicative of malnutrition risk in acute hospital patients [22].

Delirium diagnosis within 48 h of admission was established using the Confusion Assessment Method (CAM) [23]. The CAM diagnostic algorithm requires the presence of (a) acute onset or fluctuating course), (b) inattention, along with either (c) disturbed consciousness or (d) disorganised thinking. Patients exhibiting two CAM features without meeting CAM diagnostic criteria were diagnosed as having subsyndromal delirium.

Baseline cognitive performance was assessed using the locally validated Abbreviated Mental Test (AMT, range 0–10) [24]. Medical records were reviewed for a prior diagnosis of established dementia. Patients were categorized as cognitively impaired if they scored less than 8 on the AMT or had a known diagnosis of dementia.

Premorbid (defined as performance in the period up to 2 weeks prior to onset of acute illness) functional performance was assessed through interview with the patient or caregiver, to determine whether patients were independent, requiring assistance or dependent in activities of daily living (ADLs) and instrumental ADLs. We defined functional decline during hospitalization as incremental assistance in ADLs at the time of discharge compared with premorbid function.

Daily intake-output charts were reviewed for adequacy of oral intake during the admission, which was categorized as optimal if the patient consumed at least three-quarter share of each meal provided at least 50% of the time throughout the hospital stay [25].

Frailty status was assessed using the Clinical Frailty Scale (CFS) (range 1–9), with CFS scores assigned based on information on premorbid functional performance, comorbidities, cognition and symptoms of exhaustion. Patients with CFS scores of 1–3 were categorized as non-frail, 4 as pre-frail, and 5–9 as frail (with increasing frailty ranging from mild, moderate, severe, very severe and terminal) [26].

Comorbidity burden was scored using the Charlson Comorbidity Index, and severity of illness at admission was assessed using the modified Severity of Illness Index [27, 28]. The primary and additional diagnoses during admission were recorded, and hospital admissions in the year preceding index admission were captured through the electronic health records. Medication reconciliation was performed routinely at admission, with polypharmacy defined as the use of at least 5 prescription medicines.

Socio-demographic data included age, gender, ethnicity, marital status and living arrangement. Information pertaining to patient’s need for a caregiver was obtained through interview with the patient or caregiver. Caregiver burden was assessed using a single question “Do you feel strained by the caregiving situation?”, with responses of “not at all” and “not particularly” being defined as low strain and responses of “to some extent”, “much so” and “very much so” being defined as high strain [29].

**Table 1:** Definitions and measures of key variables

Variable name	Definition and Measure Used	Variable classification in analysis
Frailty	A clinical syndrome characterized by increased vulnerability to adverse health outcomes when exposed to acute stressors. Assessed using Clinical Frailty Scale (CFS, range 1–9)	Analysed as a categorical variable: non-frail (CFS1-4), mild frailty (CFS 5), moderate frailty (CFS 6), severe frailty (CFS ≥ 7).
Depression	Mood assessed using Patient Health Questionnaire-2 (PHQ-2) responses and medical records for prior history of depression	PHQ-2 and history combined to derive 4 categories: PHQ-/ History-, PHQ-/History+, PHQ+/History±, uncommunicative
Malnutrition	Nutritional status assessed using 3-minute Nutritional Screen (3MNS, range 0–9)	Binary: malnutrition (3MNS ≥ 3) vs. non-malnourished
Cognitive impairment	Operationalized as impaired performance on Abbreviated Mental Test (AMT, range 0–10), or established diagnosis of dementia from clinical records.	Binary variable: AMT and history dichotomized as cognitive impaired (AMT < 8 or history of dementia) vs. unimpaired (AMT ≥ 8, no history)
Delirium	An acute neuropsychiatric disorder characterized by inattention, global cognitive dysfunction, disturbance in consciousness, assessed using Confusion Assessment Method (CAM) with 2 core features and at least 1 of 2 other supportive features diagnostic of delirium; any 2 features not meeting diagnostic algorithm classified as subsyndromal	Analysed as a binary variable: delirium (including subsyndromal) vs. no delirium
Functional decline	Operationalized as the need for incremental assistance in activities of daily living (ADLs: feeding, toileting, dressing, bathing, walking) at discharge compared with patient’s baseline. Each ADL rated as independent, needing assistance or dependent	Analysed as a binary variable: functional decline (any ADL registering higher level of assistance relative to baseline) versus no functional decline (stable or improved)
Oral intake	Optimal intake was classified as consuming at least ¾ share of each meal ≥ 50% of all provided meals during the admission based on daily review of intake-output charts	Analysed as a binary variable: poor oral intake versus optimal intake
Comorbidity burden	Assessed using Charlson’s Comorbidity Index (CCI) with weighted CCI - low (0 points), medium (1–2 points), high (3–4 points) and very high (≥ 5 points)	Analysed as a categorical variable: low, medium, high, very high
Severity of illness	Severity of Illness Index (SII) with 4 levels provide a measure of the burden of illness and how sick a patient is while in hospital, to allow meaningful comparison across diagnostic groups.	Binary variable: mild (Level 1 or 2) versus severe (Level 3) as patients in intensive care or high dependency (Level 4) excluded from study
Geriatric syndromes	A range of multifactorial conditions that do not fit into discrete disease categories, share risk factors and commonly co-exist in older adults. Admission diagnoses categorized as geriatric syndromes include falls, delirium or cognitive impairment, and functional decline. Frailty and poor oral intake considered geriatric syndromes.	Each syndrome analysed as a binary variable (present or absent)

### Statistical analysis

#### Directed acyclic graphs

Directed acyclic graphs (DAGs) depict hypotheses and assumptions, offering transparency in the representation of causal relationships between variables for useful etiologic models. We constructed DAGs based on a priori knowledge from literature review to derive the most plausible causal diagrams for the effects of depression and malnutrition, respectively, on 30-day readmission. DAGs comprise nodes representing variables of interest and arrows representing causal associations between variables. Specifically, the nodes frailty, cognitive impairment, functional decline and malnutrition may be considered descendants (direct effects) of depression, while being associated with re-admission risk [19, 30, 31], thereby also representing mediating variables in the relationship between depression and 30-day readmission. Frailty, cognitive impairment, poor oral intake in hospital and functional decline are also descendants of malnutrition [32–35]. Paths are considered causal if all arrows point in the same direction (each variable causing the subsequent variable), while non-causal paths in which arrows do not all point in the same direction may contain confounders and/or colliders (2 arrows pointing into one variable). To identify the causal effect of the exposures (depression, malnutrition) on outcome of interest (30-day readmission), all non-causal paths must be blocked, without blocking any of the causal paths between the exposure and outcome. Thus, with a simple set of rules, the DAGs provide a clear conceptualization of confounding and enable derivation of a minimal adjustment set that blocks all backdoor paths without inadvertently opening closed pathways by conditioning on colliders, and avoid bias that may be introduced through erroneous adjustment for mediating variables [36, 37]. We used the programme DAGitty [38, 39], which applies the backtracking algorithm to derive the minimally sufficient adjustment sets that consist of the smallest number of variables necessary to account for confounding without affecting the sample size in the multiple regression analyses. The algorithm begins by identifying all covariates directly affected by the exposure. The presence of closed loops, which violate the necessary assumption of causal diagrams, will lead the programme to stop running. With an acyclic graph, the backtracking algorithm identifies all backdoor paths, and derives a potentially sufficient adjustment set with all backdoor paths blocked. If a set contains colliders, the algorithm proposes additional variables to account for collider adjustment as conditioning on a collider may open a backdoor path. The minimally sufficient adjustment set will be the set with the least covariates and all backdoor paths (bolded) blocked for estimating the total causal effects of depression and malnutrition on the outcome of 30-day readmission (Fig. 2A and B).

#### Univariate analysis and multiple logistic regression

Summary measures of baseline characteristics are presented as means (± standard deviations) and proportions. We performed univariate analyses comparing participants with and without 30-day readmission, using Pearson’s Chi square test for categorial variables and independent sample t-test for continuous variables. The association between depressive symptoms and malnutrition with other geriatric syndromes was examined using logistic regression. Multiple logistic regression was used to examine the independent risk of depressive symptoms and malnutrition on 30-day readmission, adjusted for the potential confounders identified in the minimal adjustment sets.

Complete-case analysis was restricted to participants with 30-day follow-up data. Potential selection bias arising from loss to follow up was subsequently addressed through sensitivity analyses (assuming that all losses to follow up were readmitted and non-readmitted respectively) and multiple imputation while holding the assumption of missing at random (MAR). Multivariate normal regression was used for multiple imputation with 20 data sets created. We performed alternative analysis to contrast the approach of DAG-derived minimal adjustment sets with conventional variable selection strategy that included depressive symptoms and malnutrition, alongside all covariates significantly associated with 30-day readmission in the multiple regression model. A representative covariate was chosen amongst covariates that were expected to be related, and collinearity diagnostics was performed to ensure no multi-collinearity. Age, gender and ethnicity were adjusted for a priori, irrespective of the univariate associations, in the conventional model.

Statistical analysis was performed using STATA 15.0. All statistical tests were 2-tailed, with *p*-value ≤ 0.05 considered statistically significant.

## Results

**Fig. 1:**
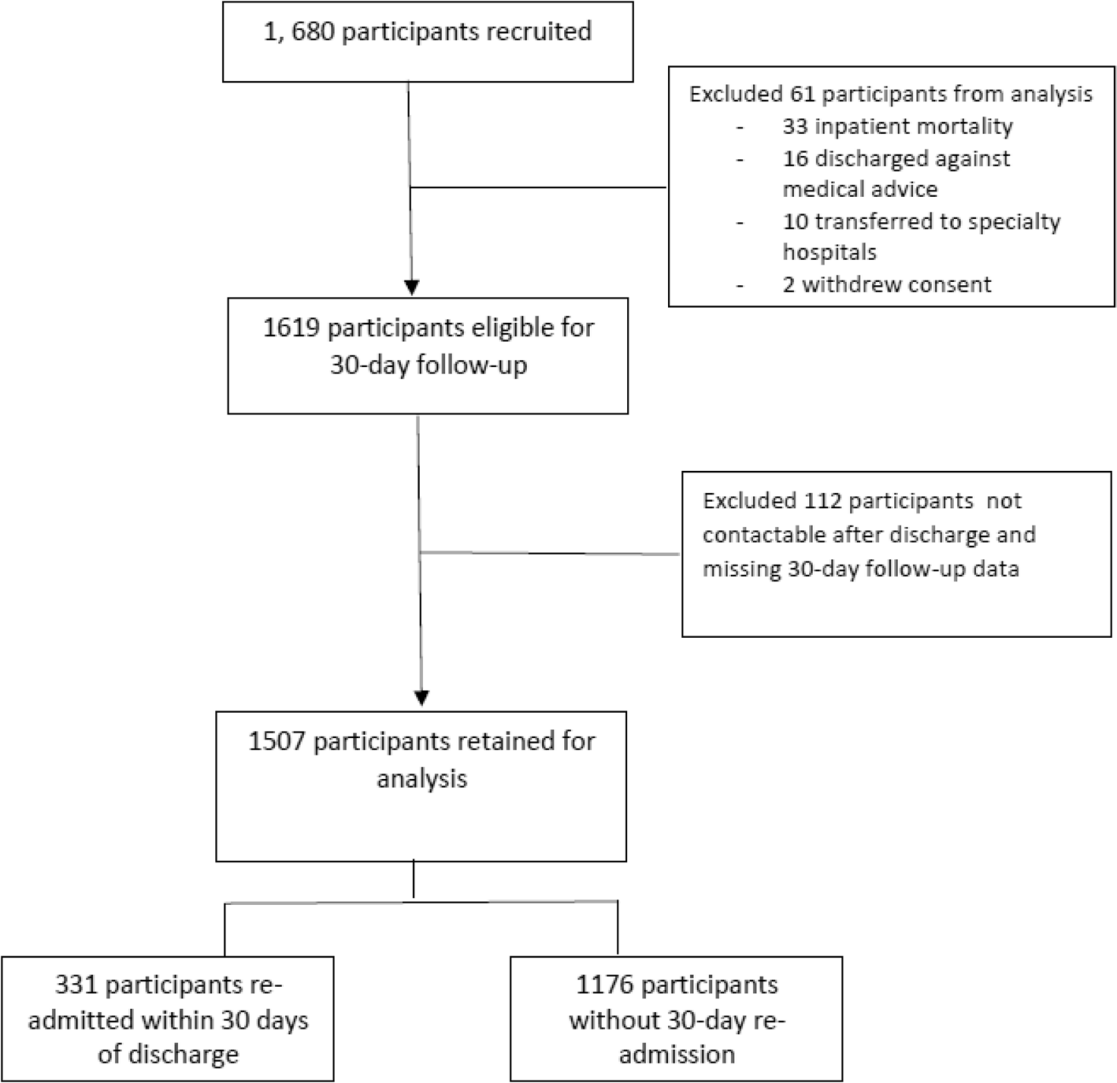
Flowchart of participants in study

 We recruited 1680 consecutive admissions, of which 1619 participants were eligible for 30-day follow-up and had a mean age 76.4 (7.9) years, with 51.3% females. 112 (6.9%) patients had missing 30-day follow-up data. Complete case analysis comparing readmitters versus non-readmitters was performed for 1507 patients, of whom 331 (22.0%) were re-admitted within 30-days of discharge. Among the eligible cohort of 1619 participants, depressive symptomatology was reported by 352 (21.7%) participants, while 44 (2.7%) participants had a history of established depression but were asymptomatic at time of admission. Malnutrition was prevalent in 13.4% participants at admission. The flowchart of participants through the study is depicted in Fig. 1.

### Factors associated with 30-day readmission

Table 2 compares the characteristics of patients with and without 30-day readmission. Patients with 30-day readmission were more likely to report depressive symptoms (26.1% vs. 20.4%, *P* < 0.001) compared with patients who were not readmitted. Malnutrition at admission was more prevalent among patients who were readmitted within 30-days compared with those without readmission (17.4% vs. 11.6%, *P* < 0.01). Patients who were readmitted within 30 days had significantly longer length of stay during the index admission compared with those who were not readmitted, with observed differences in patterns of admission diagnoses but not severity of illness. Patients with 30-day readmission had higher comorbidity burden, were more likely to have been admitted to hospital in the one-year preceding index admission, more often cognitively impaired and had more severe frailty at baseline. Delirium, functional decline and poor oral intake during the index admission were all significantly more commonly observed among patients who were readmitted within 30 days of discharge.

**Table 2:** Baseline and hospitalization characteristics for patients with and without 30-day readmission

	Readmission (*N* = 331)	No readmission (*N* = 1176)	*p* value
Socio-demographics			
Age	77.0 (8.2)	76.1 (7.7)	0.068
Gender (Female)	159 (48.0%)	606 (51.5%)	0.261
Ethnicity (Chinese)	256 (77.3%)	960 (81.6%)	0.116
Living alone	12 (3.5%)	60 (5.2%)	0.308
Admission diagnoses (top 3)			
Sepsis	110 (33.2%)	354 (30.1%)	< 0.001
Fluid overload/ ACS	30 (9.1%)	43 (3.6%)	
Geriatric syndrome^a^	42 (12.7%)	193 (16.4%)	
Severity of Illness Index			
Level 1/2	196 (59.3%)	751 (64.0%)	0.144
Level 3	134 (40.6%)	422 (36.0%)	
Length of stay (days)	6.6 (5.4)	5.6 (4.4)	< 0.001
Caregiver burden (N = 223)			
High strain	64 (42.4%)	31 (43.1%)	0.909
Comorbidity Burden			
Previous admission 1 yr	206 (62.2%)	512 (43.5%)	< 0.001
Charlson’s Comorbidity Index			< 0.001
Low	38 (11.5%)	281 (23.9%)	
Medium	128 (38.7%)	524 (44.6%)	
High	86 (26.0%)	231 (20.0%)	
Very high	79 (24.0%)	136 (11.6%)	
Polypharmacy	204 (62.2%)	550 (47.5%)	< 0.001
Baseline risk factors			
Clinical Frailty Scale			< 0.001
Non-frail (CFS 1–4)	68 (20.5%)	333 (28.3%)	
Mild frailty (CFS 5)	148 (44.7%)	565 (48.0%)	
Moderate frailty (CFS 6)	58 (17.5%)	165 (4.0%)	
Severe frailty (CFS 7/8)	57 (17.2%)	113 (9.6%)	
Depressive symptoms			< 0.001
PHQ2- / History -	195 (58.9%)	834 (71.0%)	
PHQ2- / History +	9 (2.7%)	31 (2.6%)	
PHQ2+ / History ±	86 (26.0%)	238 (20.3%)	
Uncommunicative	41 (12.4%)	71 (6.1%)	
Malnutrition	57 (17.4%)	133 (11.7%)	0.007
Cognitive impairment	112 (35.1%)	328 (28.6%)	0.025
Geriatric syndromes during admission			
Delirium (include subsyndromal)	47 (14.2%)	120 (10.2%)	0.040
Functional decline at discharge	66 (20.3%)	119 (10.1%)	< 0.001
Poor oral intake	127 (38.7%)	257 (22.2%)	< 0.001

^*ACS* Acute coronary syndrome, *PHQ *Patient Health Questionnaire^

^a^Any record of falls, delirium, cognitive impairment or functional decline in the admission diagnosis

### Association of depressive symptoms and malnutrition with geriatric syndromes

The presence of active depressive symptoms was significantly associated with frailty (CFS ≥ 5, OR = 1.62, 95% CI 1.22–2.16, *P* = 0.039), poor oral intake during hospitalization (OR = 1.35, 95% CI 1.02–1.79, *P* = 0.037), and inpatient functional decline (OR = 1.58, 95% CI 1.11–2.23, *P* = 0.011), when referenced against patients with neither depressive symptoms nor history of depression (Table 3). Prior depression diagnosis even in the absence of active depressive symptoms, as well as inability to communicate symptoms, were significantly associated with frailty, poor oral intake, functional decline, delirium and cognitive impairment. Malnutrition was significantly associated with all geriatric syndromes examined, with the exception of functional decline during hospitalization (*P* = 0.076).

**Table 3:** Univariate logistic regression models for depressive symptoms and malnutrition on geriatric syndromes

	Frailty (CFS ≥ 5)	Delirium	Cognitive impairment	Poor oral intake	Functional decline
Depressive symptoms					
PHQ2- / History -	Ref	Ref	Ref	Ref	Ref
PHQ2- / History +	2.36* (1.04–5.35)	4.73* (2.24-10.00)	2.07* (1.11–3.87)	2.18* (1.16–4.11)	2.22* (1.04–4.74)
PHQ2+ / History ±	1.62* (1.22–2.16)	1.18 (0.73–1.91)	0.85 (0.64–1.13)	1.35* (1.02–1.79)	1.58* (1.11–2.23)
Uncommunicative	27.72* (6.82-112.74)	35.45* (22.50-55.88)	73.56* (23.06-234.82)	10.76* (7.00-16.53)	3.45* (2.24–5.33)
Malnutrition	1.53* (1.07–2.19)	2.33* (1.60–3.39)	1.88* (1.39–2.54)	2.70* (2.01–3.64)	1.43 (0.96–2.11)

Separate univariate logistic regression models were performed with depressive symptoms and malnutrition each as independent variables, on individual outcomes of frailty, delirium, cognitive impairment, poor oral intake and functional decline

*PHQ* Patient Health Questionnaire

*P < 0.05

### DAG-derived adjustment for the causal association of depressive symptoms and malnutrition with 30-day readmission

**Fig. 2 A:**
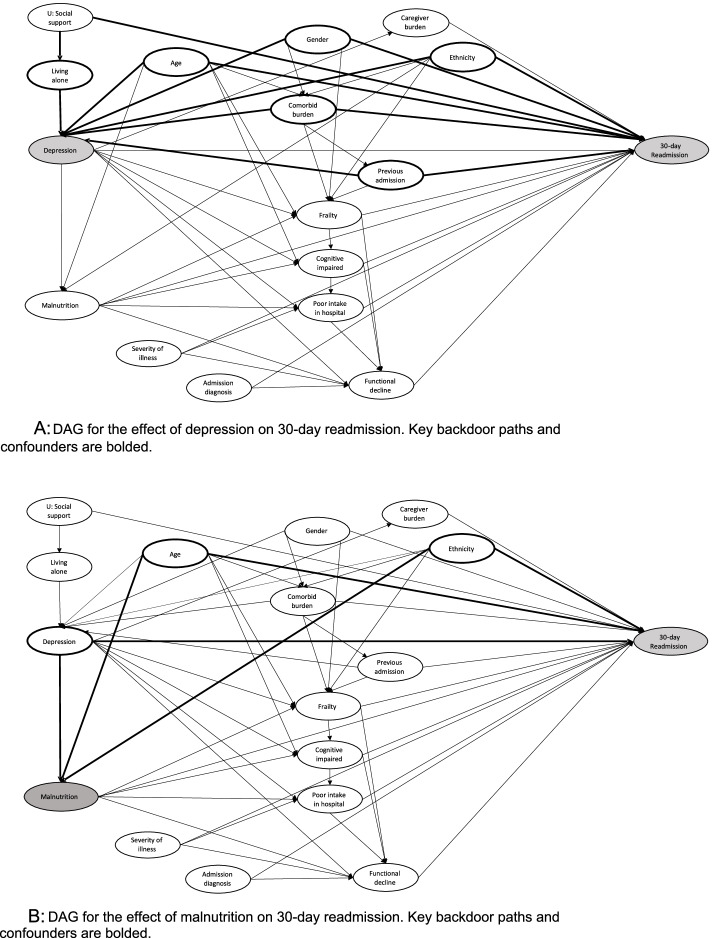
DAG for the effect of depression on 30-day readmission. Key backdoor paths and confounders are bolded. **B** DAG for the effect of malnutrition on 30-day readmission. Key backdoor paths and confounders are bolded

For the effect of depression on 30-day readmission with 15 co-variates, 29 causal paths were identified and the minimal adjustment set included age, comorbidity, gender, ethnicity, living alone and having an admission in the preceding one year (Fig. 2A). After adjusting for the confounding variables in the minimal adjustment set, patients reporting depressive symptoms had significantly increased risk for 30-day readmission (OR = 1.38, 95% confidence interval 1.02–1.86, *P* = 0.039) when referenced against patients with neither depressive symptoms nor history of depression (Table 4). Patients with an established depression diagnosis but not experiencing active depressive symptoms were not at risk for 30-day readmission. Patients who were unable to communicate their symptoms were also at increased risk for 30-day readmission (OR = 2.11, 95% confidence interval 1.35–3.28, *P* = 0.001). Positive depressive symptoms and inability to communicate depressive symptoms remained significant risk factors for 30-day readmission in multiple imputation and sensitivity analyses (Supplementary Table).

The DAG for malnutrition on 30-day readmission included 14 causal paths and age, depression and ethnicity were identified as potential confounders in the minimal adjustment set (Fig. 2B). Malnutrition at admission was significantly associated with 30-day readmission in the unadjusted model (OR = 1.59, 95% confidence interval 1.14–2.230, *P* = 0.007). In the minimal adjustment set to account for confounding by age, ethnicity and depressive symptoms, malnutrition retained association with 30-day readmission, although no longer statistically significant (OR = 1.40, 95% confidence interval 0.99–1.98, *P* = 0.058) (Table 4). Malnutrition remained significantly associated with 30-day readmission in multiple imputation analysis (OR = 1.66, 95% confidence interval 1.35–2.05, *p* < 0.001), and in sensitivity analyses for which all patients with missing 30-day follow up were assumed as having been readmitted (OR = 1.48, 95% confidence interval 1.08–2.02, *P* = 0.014). The association between malnutrition and 30-day readmission was no longer statistically significant in sensitivity analyses that assumed missing follow-up data as not having been readmitted (Supplementary Table).

**Table 4:** Estimated odds ratios for depressive symptoms and malnutrition on 30-day readmission

	Model 1, Odds Ratio (95% CI)	Model 2 Odds Ratio (95% CI)	Model 3 Odds Ratio (95% CI)
Depressive symptoms			
PHQ2- / History –	Ref	Ref	Ref
PHQ2- / History +	1.24 (0.58–2.65) *P* = 0.576	1.03 (0.47–2.24), *P* = 0.947	0.98 (0.44–2.19) *P* = 0.961
PHQ2+ / History ±	1.55 (1.15–2.07), *P* = 0.003	1.38 (1.02–1.86), *P* = 0.039	1.32 (0.96–1.80) *P* = 0.087
Non-communicative	2.47 (1.63–3.74), *P* < 0.001	2.11 (1.35–3.28), *P* = 0.001	2.00 (1.13–3.58) *P* = 0.018
Malnutrition	1.59 (1.14–2.23), *P* = 0.007	1.40 (0.99–1.98), *P* = 0.058	1.17 (0.81–1.69) *P* = 0.414

*PHQ* Patient Health Questionnaire

Model 1: Unadjusted univariate logistic regression of depressive symptoms and malnutrition, separately, on 30-day readmission

Model 2: DAG-based minimal adjustment set. The model for depressive symptoms included age, gender, ethnicity, comorbidity burden, living alone and admission in preceding one year. The model for malnutrition included age, ethnicity, and depressive symptoms

Model 3: Conventional model including depressive symptoms and malnutrition, adjusted for admission in the preceding one year, admission diagnosis, length of stay, comorbidity burden, frailty, delirium, functional decline at discharge, poor oral intake, age, gender, ethnicity

Using the conventional approach to examine the independent effects of depression and malnutrition on 30-day readmission in multiple logistic regression that adjusted for variables with univariate *P* < 0.05 - admission in the preceding one year, admission diagnosis, length of stay, comorbidity burden, frailty, delirium, functional decline at discharge, poor oral intake - alongside a priori adjustment for age, gender and ethnicity, depressive symptomatology and malnutrition were no longer associated with readmission risk, although patients who were unable to communicate symptoms remained at increased risk for 30-day readmission (Table 4).

## Discussion

Our study identified a 30-day readmission rate of 22%, and established the causal associations of depressive symptoms and malnutrition with 30-day readmission among hospitalized older adults. Additionally, both depressive symptoms and malnutrition at admission were associated with other geriatric syndromes, including delirium, cognitive impairment, frailty, poor oral intake and functional decline during hospitalization.

Our working DAGs and associations between variables of interest were guided by a priori knowledge from literature review. DAGs offer the advantage of clearly depicting the confounders and potential biases in exposure-outcome relationships through simple graphical representation, and avoid inadvertent overadjustment that may be introduced through adjusting for mediators in the causal paths between exposure and outcome. This ensures only common causes (confounders) are adjusted for in our multiple logistic regression models, without inappropriate adjustment for mediators that would attenuate or even reverse the true causal effect of exposure [37]. As illustrated in Fig. 2A and B, frailty [18, 19], cognitive impairment [40], functional decline [41] and poor oral intake during admission would have been descendants of depressive symptoms and malnutrition, and thereby considered mediators in the causal paths between depressive symptoms or malnutrition and 30-day readmission. In conventional analyses that adjusted for these mediators, we failed to observe associations between depressive symptoms and malnutrition on readmission, plausibly due to bias introduced by overadjustment. However, DAGs are purely qualitative and do not convey the magnitude of effect measure modification. As our DAGs were constructed on hypothesized relationships based on prior literature review, future studies could consider focusing on the mechanisms by which depression and malnutrition aggravate readmission risk. Additionally, mediation analysis may also deepen our understanding of the factors at play in driving readmission in hospitalized older adults through direct and indirect effects, with the insights gained facilitating more efficient intervention strategies.

Earlier studies that reported association of depression with readmission risk had operationalized depression based on documentation in patient’s record of a diagnosis, history of depression using International Classification of Diseases (ICD-10) diagnosis codes, psychiatric diagnosis or filling of antidepressant prescription [42, 43]. Using quick screening questions for depressive symptoms in the PHQ-2 [21], and further categorization based on established depression diagnosis, our findings suggest that active symptomatology may provide greater clinical relevance in identifying older adults at risk for readmission, compared with the mere dependence on historical diagnosis of depression. A prior history of depression in the absence of active depressive symptoms was not associated with readmission risk, corroborating an earlier study in which ongoing symptoms as identified using PHQ-9 but not depression history predicted short-term mortality or readmission following discharge. There was also only modest correlation between symptoms and prior history, suggesting that a reliance on historical diagnosis may not offer adequate substitute for symptomatic depression which confers greater prognostic significance [44]. Subsyndromal depression is common in older adults, and imposes the same clinical significance and health impact as syndromal depression, and should be accorded due attention and intervention [45]. Contrary to an earlier study which reported no significant association between depressive symptoms and 30-day readmission in hospitalized older adults [46], cognitive impairment did not preclude study participation in our cohort. Indeed, depressive symptoms are common in patients with dementia and contribute to excess morbidity and mortality, yet are often poorly recognized as patients with dementia may be unable to verbalize feelings of sadness [47]. The increased risk for readmission among patients who were unable to communicate symptoms was notably higher than that for patients reporting depressive symptoms. Our approach of differentiating patients who were unable to communicate symptoms contrasts with a recent study classifying aphasic patients as being positive for depression which was also linked to increased risk for readmission [13]. The inability to communicate can be a manifestation of severity of underlying dementia or delirium, which may be driving the readmission risk, as observed in our examined associations between depressive symptoms and the various geriatric syndromes. However, depression remains highly prevalent across dementia stages, and may present as behavioural disturbance such as agitation, sleep disturbance, decreased appetite or withdrawal in persons with advanced dementia who are unable to verbalize symptoms [48, 49]. Owing to the inconsistent documentation practices of such behavioural manifestations, we were unable to confidently ascertain the potential for depression amongst our less communicative patients.

Recent studies highlighted malnutrition among the geriatric syndromes contributing to readmission risk in older adults, and nutritional problem was the strongest predictor identified from a Comprehensive Geriatric Assessment [12, 13]. Our observed association between malnutrition and 30-day readmission justify efforts at targeted nutritional interventions for malnourished hospitalized older adults. Malnutrition was also associated with other geriatric syndromes and perpetuated poor oral intake during hospitalization. Thus, early identification and supportive dietary strategies will be paramount to address a potential vicious cycle driving readmission risk. With literature evidence suggesting depression as a contributing cause of malnutrition [50], possibly owing to reasons such as inappetence, loss of interest in self-care and apathy, our working DAG specified depression as a parent of malnutrition, and subsequently addressed as a confounding variable in the relationship between malnutrition and 30-day readmission. In DAG-based analysis, the effect of malnutrition on 30-day readmission was attenuated once depressive symptoms were adjusted for, although significant association was maintained in multiple imputation and sensitivity analyses. Comparatively, we hypothesized that the observed associations between comorbidity burden and malnutrition in many cross-sectional studies are driven through depression, thereby guiding the direction of arrows in our working DAG. This was supported by the results from a study in frail older adults, in which depression rather than comorbidity index was associated with malnutrition in multiple regression analyses [51]. This contrasts with earlier approaches which typically adjusted for comorbidities, admission diagnoses and length of hospital stay in examining the association between nutritional status and readmission, which in alternative analyses using the traditional approach for selection of variables would have nullified the association between malnutrition and 30-day readmission in our cohort.

A review of existing literature suggests that this is the first study adopting DAGs to examine the relationship between modifiable risk factors and 30-day readmission in older adults. Utilizing DAGs allows visualization and analysis of complex causal situations, such as in the case of unplanned hospital readmissions. Readmissions are typically multifactorial and complex events, with socioeconomic, medical and psychological factors typically being implicated. The DAG offers transparency in the representation of causal relations between variables based on knowledge, theories or assumptions. This provides a valid model for the identification of an implied adjustment set to accurately estimate a causal effect through inspection or algorithmically, depending on the DAG’s structure and complexity. However, we acknowledge several limitations in our study, in particular the exclusion of patients who were unable to provide informed consent and had no legally acceptable representative who could be approached for this study, leading to possible selection bias and limiting generalizability of our study findings to all hospitalized older adults. We would like to highlight that cognitively impaired patients were still represented and accounted for approximately 30% of our cohort, although only 3% were severely impaired and unable to communicate. While our analysis was restricted to patients with complete follow-up data at 30-days, subsequent sensitivity and multiple imputation analyses supported the results of the complete-case analysis, although not addressing the competing risk of mortality.

Our findings are generalizable to older adults admitted to a medical unit, and were community-dwelling prior to and after the acute hospital stay. Extrapolation beyond the study setting requires cautious interpretation as the readmission problem in surgical and institutionalized patients may be fundamentally different. Future studies could consider validation of the current findings in non-medical cohorts, while further exploring whether the addition of potentially modifiable risk factors such as geriatric syndromes improve the performance of predictive models for hospital readmission in older adults.

## Conclusion

In conclusion, depressive symptoms and malnutrition are associated with other geriatric syndromes, and represent potentially modifiable risk factors for 30-day readmission in hospitalized older adults. The results support efforts to improve screening and identification of active depressive symptoms and malnutrition for targeted interventions during admission and in the post-acute period.

## Supplementary Information

**Additional file 1: Supplementary Table 1.** Sensitivity Analyses for Depressive Symptoms on 30-day Readmission. **Supplementary Table 2.** Sensitivity Analyses for Malnutrition on 30-day Readmission.

## Data Availability

All data analysed during this study are included in this published article.
