# Supplementary material for: Depressive symptoms and malnutrition are associated with other geriatric syndromes and increase risk for 30-Day readmission in hospitalized older adults: a prospective cohort study
Source: BMC Geriatr. 2022 Aug 2;22:634. doi: 10.1186/s12877-022-03343-6 (PMC9344637; doi:10.1186/s12877-022-03343-6)
Supplement: Supplementary file 1 — Additional file 1: Supplementary Table 1. Sensitivity Analyses for Depressive Symptoms on 30-day Readmission. Supplementary Table 2. Sensitivity Analyses for Malnutrition on 30-day Readmission. [file 12877_2022_3343_MOESM1_ESM.docx]

Supplementary Table 1: Sensitivity Analyses for Depressive Symptoms on 30-day Readmission

|  | Model 1  Odds Ratio (95% CI) | Model 2  Odds Ratio (95% CI) | Model 3  Odds Ratio (95% CI) |
| --- | --- | --- | --- |
| Depressive symptoms  PHQ2- / History –  PHQ2- / History +  PHQ2+ / History +  Non-communicative | Ref  1.07 (0.54-2.12)  P=0.840  1.42 (1.09-1.87)  P=0.010  2.12 (1.42-3.16)  P<0.001 | Ref  0.93 (0.43-2.02)  P=0.861  1.35 (1.00-1.82)  P=0.048  1.88 (1.22-2.88)  P=0.004 | Ref  1.17 (0.78-1.80)  P=0.477  1.57 (1.32-1.87)  P<0.001  2.05 (1.55-2.71)  P<0.001 |

Model 1: Assuming all missing data readmitted; DAG-based minimal adjustment set including age, gender, ethnicity, comorbidity burden, living alone and admission in preceding one year

Model 2: Assuming all missing data not readmitted; DAG-based minimal adjustment set including age, gender, ethnicity, comorbidity burden, living alone and admission in preceding one year

Model 3: Multiple imputation analysis; DAG-based minimal adjustment set including age, gender, ethnicity, comorbidity burden, living alone and admission in preceding one year

Supplementary Table 2: Sensitivity Analyses for Malnutrition on 30-day Readmission

|  | Model 1,  Odds Ratio (95% CI) | Model 2  Odds Ratio (95% CI) | Model 3  Odds Ratio (95% CI) |
| --- | --- | --- | --- |
| Malnutrition | 1.48 (1.08-2.02),  P=0.014 | 1.34 (0.95-1.88)  P=0.096 | 1.66 (1.35-2.05)  P<0.001 |

Model 1: Assuming all missing data readmitted; DAG-based minimal adjustment set including age, ethnicity and depressive symptoms

Model 2: Assuming all missing data not readmitted; DAG-based minimal adjustment set including age, ethnicity and depressive symptoms

Model 4: Multiple imputation analysis; DAG-based minimal adjustment set including age, ethnicity and depressive symptoms
